# A modular Uba1-nanobody fusion enables selective ubiquitin transfer to tagged E2 enzymes

**DOI:** 10.1016/j.jbc.2025.110910

**Published:** 2025-11-05

**Authors:** Charlotte Wijne, Francesca D'Amico, David A. Pérez Berrocal, Monique P.C. Mulder, Hidde L. Ploegh, Jin Gan

**Affiliations:** 1 Program in Cellular and Molecular Medicine, Boston Children's Hospital, Harvard Medical School, Boston, Massachusetts, USA; 2 Department of Cell and Chemical Biology, Leiden University Medical Center, Leiden, The Netherlands

**Keywords:** ubiquitin, UBA1, ubiquitin E2, nanobody, ubiquitination, Ubiquitin-dehydroalanine probe

## Abstract

The ubiquitin-activating enzyme Uba1 initiates the ubiquitination cascade by activating ubiquitin and subsequently transferring it to a broad range of E2 conjugating enzymes *via* its C-terminal ubiquitin-fold domain. Tools to selectively redirect this transfer to defined E2s, and thus govern control of E2 enzyme usage, are limited. By replacing the ubiquitin-fold domain of Uba1 with the high-affinity nanobody VHH05, we create an engineered E1 enzyme (Uba1-VHH05) that selectively engages E2s fused to the 6e-tag, which is the epitope recognized by VHH05. This plug-and-play interface preserves native Uba1 catalytic activity while allowing ubiquitin loading to be directed toward user-defined E2s without altering other components of the cascade. We show that Uba1-VHH05 supports transfer of both WT ubiquitin and the activity-based probe Ub-Dha to a range of tagged E2s. This recapitulates endogenous *in vitro* activities, including polyubiquitin chain formation. This strategy enables precise dissection of E2-specific functions and offers a new tool to generate orthogonal ubiquitin cascades *in vitro* and ultimately in cells.

Ubiquitination is an essential and versatile post-translational modification that governs nearly every aspect of normal cellular function including DNA repair, cell cycle progression, signal transduction, autophagy, and protein degradation *via* the ubiquitin-proteasome system (1, 2, 3, 4, 5, 6, 7, 8, 9, 10). The attachment of ubiquitin (Ub) to substrate proteins occurs through a three-step enzymatic cascade involving a ubiquitin-activating enzyme (E1), a ubiquitin-conjugating enzyme (E2) and finally a ubiquitin ligase (E3) (11, 12). In humans, two E1 enzymes initiate the cascade by activating ubiquitin, then delivering it into a network of ∼35 active E2s and over 600 E3 ligases (13, 14, 15). Although E3 enzymes are considered to be the main arbiters of substrate specificity, interactions among E1, E2, and E3 enzymes collectively shape efficiency, linkage type, and selectivity of ubiquitin transfer (16, 17, 18). These layers of regulation are critical to maintain cellular homeostasis, and when disrupted contribute to disease ranging from neurodegeneration to cancer (19, 20, 21, 22).

Of the two human E1 enzymes, Uba1 is the primary initiator of ubiquitination across most cellular contexts (23). Uba1 is a large, multidomain protein (Fig. 1, *A* and *D*) that must coordinate ATP- and ubiquitin-binding, ubiquitin activation as well as ubiquitin transfer to a E2 enzyme—tasks that demand tight conformational control (16, 24). Ubiquitin activation begins at the active and inactive adenylation domains (AAD and IAD), where a ubiquitin-AMP intermediate is formed in the presence of ATP and Mg^2+^ (25, 26, 27). This intermediate is then transferred to the catalytic cysteine domain, composed of the first and second catalytic cysteine half-domains (FCCH and SCCH), where a thioester bond is formed between the active-site cysteine of Uba1 and the C-terminal glycine of ubiquitin (17, 28). Uba1 next engages the E2 *via* its C-terminal ubiquitin-fold domain (UFD), which recognizes a conserved basic N-terminal motif on the E2. This UFD-E2 engagement induces a conformational rearrangement that allows the catalytic cysteines of both enzymes to align, which enables the transthiolation of ubiquitin to the E2 and allows the continuation of the ubiquitin cascade (29, 30, 31, 32). Although the UFD–E2 interface shows a degree of plasticity across different E2s, the juxtaposition of the two catalytic cysteines is highly conserved. This layered recognition mechanism allows Uba1 to accommodate a broad range of E2s while maintaining precise thioester transfer. In this work, we exploit Uba1’s conformational flexibility required to enable both Ub activation and Ub transfer through proper positioning of the UFD domain, as will be described below.

**Figure 1:**
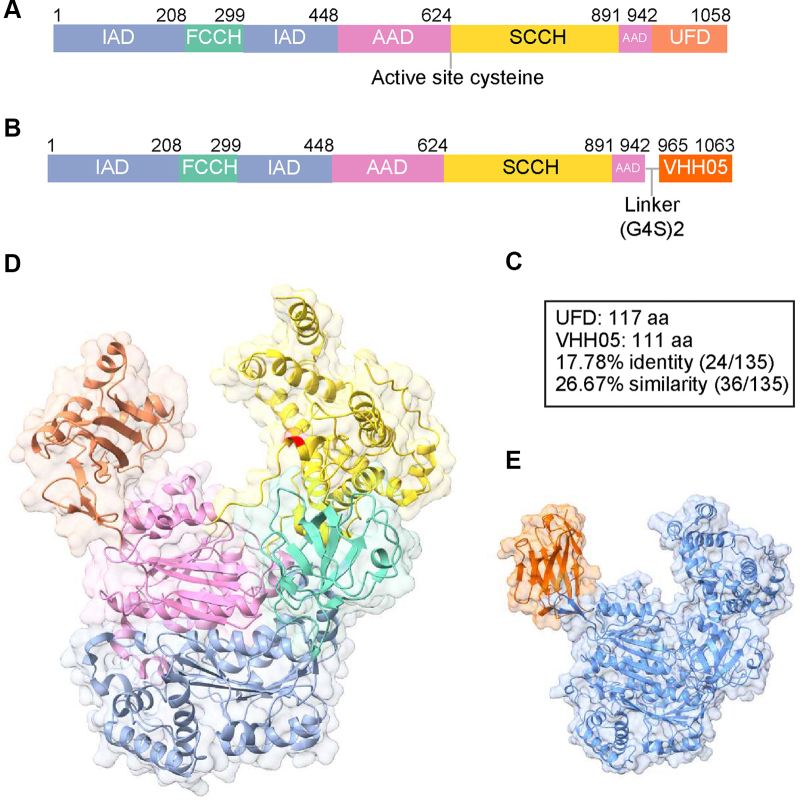
**Replacement of the Uba1 ubiquitin-fold domain (UFD) with VHH05.***A*, domain organization overview for human Uba1. The five domains are annotated and color coded, corresponding residue numbers are listed above. The active site cysteine at position 632 is annotated below. *B*, domain overview for the Uba1 where the UFD domain is replaced with VHH05. *C*, domain comparison between UFD and VHH05, with identity and similarity percentages, as calculated by Needleman-Wunsch analysis. *D*, the resolved structure of human Uba1 (PDB: 6DC6) with the functional domains color coded as in (*A*). The active site cysteine is highlighted in *red* within the SCCH domain. *E*, the AlphaFold3 prediction for the Uba1-VHH05 construct. The VHH05 domain is colored *orange*, and all remaining domains have been colored *blue*. The orientation of the predicted structure is identical to Uba1 WT in (*D*). Structural prediction parameters can be found in Fig. S1. PDB, Protein Data Bank.

Despite the central role of Uba1-E2 interactions, few tools exist to manipulate these steps with precision. Current strategies mostly focus on global blocking of Uba1 activity. For example, the small molecule TAK-243 blocks Uba1’s adenylation activity, thereby preventing ubiquitin activation (33, 34, 35). While effective, such approaches indiscriminately shut down ubiquitination and do not enable reprogramming of specific E1–E2 interactions. Orthogonal E1–E2 cascade systems, such as those developed by Zhao *et al.*, demonstrate that engineered interfaces can redirect ubiquitin transfer with high specificity (36). However, because these systems require full co-engineering of all cascade components and operate independently from endogenous ubiquitin signaling, they are less suited for interrogating endogenous E2 function. More recently, methods such tCubE and E2∼dID have enabled E2-specific substrate profiling in cells and lysates, but these rely on extensive and complex construct design and artificial conjugation strategies. Moreover, they bypass the native E1-E2 transfer step as well as limit E3 usage (37, 38). Thus, there is a need for new methods that allow precise control over which E2s are loaded by Uba1, while preserving the integrity of the ubiquitin cascade. Such tools would ultimately enable the dissection of E2-specific ubiquitination events in a native cellular context, and uncover how individual E2s influence chain type, E3 recruitment, and substrate selection. The work described here presents proof of concept that these goals may be attainable.

To monitor active intermediates in the ubiquitin cascade, Mulder *et al.* developed a covalent activity-based probe, in which the C-terminal glycine of ubiquitin was replaced by a reactive dehydroalanine residue (Ub-Dha) (39). Upon activation by Uba1, the Ub-Dha probe participates in the ubiquitin cascade in two distinct ways: it either forms a transient thioester bond with the enzyme’s active-site cysteine and thus mimics native ubiquitin intermediates (Fig. S3*B*, top panel), or it forms irreversible thioether linkages that covalently trap the enzyme (Fig. S3*B*, bottom panel). In this way, the probe mimics native ubiquitin activation and transfer steps (Fig. S3*A*) while also enabling the stable capture of engaged catalytic intermediates (39, 40). Ub-Dha has been used to label and isolate active components of the ubiquitination machinery both *in vitro* and in cells (39, 41). However, because RING E3s act as scaffolds for ubiquitin transfer from E2 to substrate rather than catalytic partners, this approach provides limited information on RING-type E3 engagement. In addition, the Ub-Dha probe cannot be transferred to substrates. Complementary approaches are therefore needed to selectively direct and study ubiquitination through specific E2 enzymes.

Here, we report the development of a novel system to study E2-specific ubiquitination in which the UFD domain of Uba1 is replaced with the anti-Ube2J1 nanobody VHH05 (Uba1-VHH05). The VHH05 nanobody binds with high affinity to a 14-amino acid epitope known as the 6e-tag (42). By fusing the 6e-tag to the E2 of interest, we create an E1–E2 interface that enables selective loading of a single, 6e-tagged E2 by the VHH05-engineered Uba1. Using this system, we show that Uba1-VHH05 can successfully be loaded with either Ub-Dha or WT ubiquitin and that this construct can selectively transfer either form of ubiquitin to tagged E2s. Thus, this design preserves Uba1's normal activity but alters its mode of E2 engagement. This makes it possible to direct ubiquitin transfer specifically through a user-defined E2. Because the same engineered Uba1 can be used in combination with different tagged E2s, this platform offers a broadly applicable and scalable approach for interrogating the roles of individual E2s in ubiquitination. The system proposed here could thus be a useful tool to dissect E2-specific contributions to ubiquitin signaling in a native biochemical context.

## Results

### The UFD domain of Uba1 can be successfully replaced with a VHH

To generate Uba1 variants with E2-selective loading capabilities, we replaced the native UFD of Uba1 with one of two nanobodies: VHH05, which recognizes a 14-amino acid epitope known as the 6e-tag, and Nb127D01, a control nanobody with no known affinity for E2s (42, 43). These chimeric Uba1 constructs are referred to as Uba1-VHH05 and Uba1-Nb127D01, respectively. A comparative sequence overview for the WT Uba1 and Uba1-VHH05 is given in Figure 1, *A* and *B*. Importantly, the size of the nanobody (∼15 kDa) is comparable to that of the native UFD (Fig. 1*C*), suggesting that the UFD replacement should not be grossly disruptive to the overall architecture of Uba1. Two repeats of a G4S linker were inserted between the Uba1 core and the appended VHH05 to allow for flexibility between them.

To assess whether our nanobody replacement strategy perturbs the overall Uba1 structure, we used AlphaFold3 to model the full-length Uba1 construct in which the UFD was replaced with the VHH05 nanobody (Figs. 1*E* and S1*A*) (44). The model showed high overall confidence (pTM = 0.86), with well-ordered folding across all structured domains. We next compared the predicted Uba1-VHH05 structure to the crystal structure of WT Uba1 (Protein Data Bank [PDB] 6DC6) (Figs. 1, *D*, *E* and S1*B*) (45). Structural alignment of residues 49 to 954 yielded a low RMSD of 0.617 Å, indicating that substitution of the UFD with VHH05 does not induce major conformational changes in the Uba1 core.

In parallel, we generated a series of E2 enzymes tagged with the 6e-tag to enable select engagement by the Uba1-VHH05. The 6e-tag was fused C terminally to Ube2R1, Ube2T, and Ube2B as well as N terminally to Ube2B (Fig. S2). All constructs were cloned, expressed in *Escherichia coli*, and purified as described in the “Experimental procedures” section.

### Uba1 UFD-mutants can be successfully charged with Ub-Dha probe

To evaluate whether Uba1 constructs bearing nanobody substitutions remained catalytically competent, we tested their ability to be loaded with the Ub-Dha probe. This probe can form both a thioester- as well as a stable thioether-adduct with the active-site cysteine of E1 enzymes, effectively capturing the normally transient intermediate in a covalent and irreversible manner (39). This approach allowed us to directly assess the formation of the E1∼Ub intermediate without the risk of hydrolysis during sample preparation or electrophoresis.

**Figure 2:**
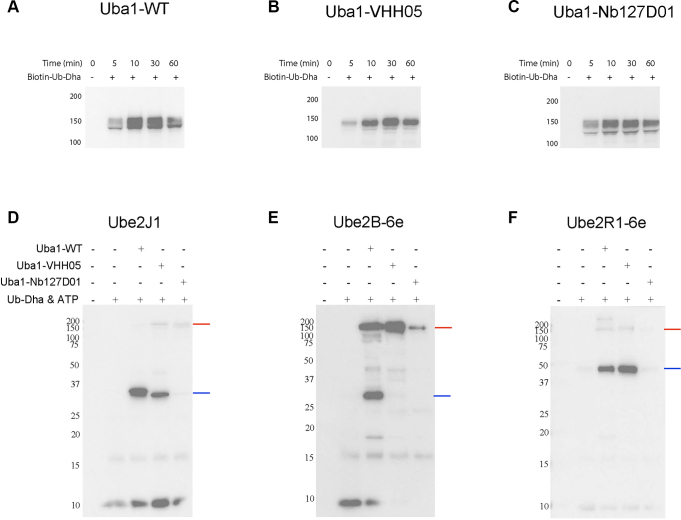
**Loading of Ub-Dha on Uba1 UFD-replacement variants.***A*–*C*, loading efficiency of the ubiquitin dehydroalanine (Ub-Dha) probe on to the different UFD-replacement Uba1 constructs was evaluated by coincubation of the Uba1-variants with biotinylated Ub-Dha probe. The immunoblots for WT Uba1 (*A*) and the variants where the UFD domain was replaced by VHH05 (*B*) or Nb127D01 (*C*) demonstrate efficient probe charging onto these constructs. *D*–*F*, Uba1-VHH05 selectively transfers the Ub-Dha probe to Ube2J1, which contains the 6e-tag within its sequence, (*D*) and Ube2R1-6e (*E*) but not to Ube2B-6e (*F*), indicating that successful E2 engagement depends on nanobody-epitope recognition. *Red arrows* indicate E1∼UbDha conjugates and *blue arrows* mark E2∼UbDha species. Streptavidin immunoblots are shown; see Experimental procedures for reaction conditions. UFD, ubiquitin-fold domain.

We incubated WT Uba1, Uba1-VHH05, and Uba1-Nb127D01 with biotinylated Ub-Dha under standard charging conditions. Samples were collected after 0, 5, 10, 30, and 60 min of incubation at 37 °C and added to nonreducing sample buffer. Samples were analyzed by streptavidin immunoblotting (Fig. 2, *A*–*C*). Membranes were stained with Ponceau S prior to blotting to assess equal loading (Fig. S5). All three constructs formed Ub-Dha conjugates over time, with charging largely complete by 10 min for all variants. Although overall conjugation profiles were comparable, we observed subtle differences in the migration pattern and band intensity of Uba1-VHH05 relative to Uba1-WT/Nb127D01. Specifically, Uba1-VHH05 (Fig. 2*B*) appeared as a single band, lacking the small lower band present in the other samples (Fig. 2, *A* and *C*). A slightly stronger band was observed for Uba1-VHH05 at the 30-min time point, but this effect was not sustained at 60 min. These differences may reflect minor variation in protein purity, gel migration or protein loading. However, taken together, these results indicate that UFD replacement with a nanobody does not grossly impair Uba1’s ability to engage ubiquitin or form a covalent intermediate with Ub-Dha.

### Uba1-VHH05 can successfully transfer the Ub-Dha probe to 6e-tag containing E2s

Having established that all Uba1 variants could be loaded with Ub-Dha, we next tested their ability to transfer the probe to downstream E2 enzymes. For this analysis, we included Ube2J1, which naturally contains the 6e-tag within its sequence, as well as C terminally 6e-tagged Ube2R1 and Ube2B. Reactions containing one of three Uba1 variants, a 6e-tag containing E2, biotinylated Ub-Dha, and ATP were incubated at 37 °C for 1 h. Samples lacking both Ub-Dha, ATP, and Uba1, as well as reactions omitting only Uba1, were included as negative controls.

As expected Uba1-WT successfully transferred Ub-Dha to all E2s tested, as detected by streptavidin immunoblotting and indicated by the presence of E2-Ub adducts (Fig. 2, *D*–*F*, lane 3, *blue arrows*). No E2-Ub adducts were observed in any of the negative control conditions (Fig. 2, *D*–*F*, lanes 1 and 2), confirming the requirement of all components for Ub transfer. Importantly, both Ube2J1 and Ube2R1-6e formed E2-UbDha adducts when incubated with Uba1-VHH05 (Fig. 2, *D* and *F*, lane 4) demonstrating successful E2 engagement *via* nanobody–epitope recognition. In contrast, Uba1-Nb127D01 failed to transfer Ub-Dha to any E2 (Fig. 2, *D*–*F*, lane 5), but did support its own modification by the probe, indicating that the absence of transfer reflects a failure in E2 engagement and not catalytic incompetence.

No Ub-Dha transfer was observed to Ube2B-6e under any conditions aside from Uba1-WT, despite clear evidence of probe loading onto Uba1-VHH05 (Fig. 2*E*). This result suggests that Ube2B may require additional interaction motifs or a more native context for efficient E1–E2 engagement and transfer.

To further optimize this construct, we explored whether the insertion of a flexible G4S linker between the Uba1 core and the VHH05 nanobody had any effect on functionality compared to a construct that had no flexible linker present (Uba1-VHH05) (Fig. S4). Insertion of a flexible G4S linker in any capacity greatly improved Ub-Dha loading efficiency on both Uba1 and Ube2J1, which naturally contains the 6e-tag sequence, but no substantial differences were seen between constructs containing one, two, or three G4S repeats. This suggests that while linker flexibility is important for activity, the precise length is not a major determinant.

### Uba1-VHH05 supports transfer of WT ubiquitin to an array of 6e-tagged E2 enzymes

Encouraged by results obtained with the Ub-Dha probe, we next evaluated whether the Uba1 variants supported ubiquitin transfer under more physiologically relevant conditions. To this end, we performed ubiquitin-loading assays using WT ubiquitin. To test the broader applicability of our system, we included a larger panel of 6e-tagged E2 enzymes: Ube2J1, Ube2R1, Ube2T, and Ube2B. Because UFD interactions with E2s under native conditions often depend on the flexible N-terminal tail of the E2, we included both N and C terminally tagged versions of Ube2B to assess whether positioning of the tag influences recognition by Uba1-VHH05. Untagged Ube2R1 and Ube2B were included as negative controls. Reactions were incubated at 37 °C, and samples were collected at 0 and 1 h.

**Figure 3:**
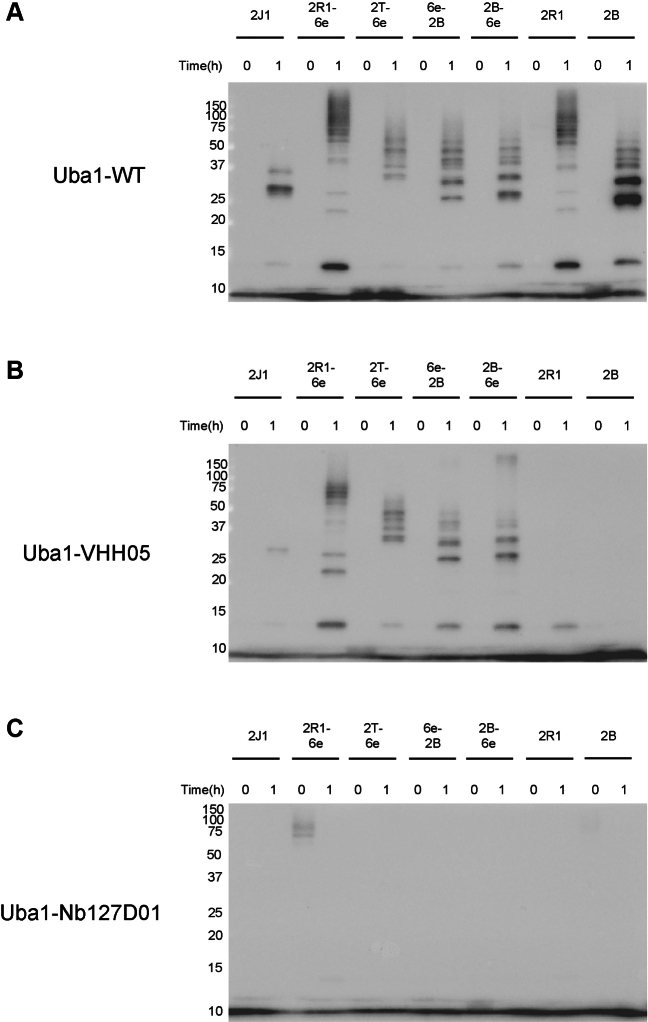
**Uba1-VHH05 can load ubiquitin onto Ube2J1 and other 6e-tagged constructs.***In vitro* ubiquitin loading assay (anti-ubiquitin immunoblot) demonstrating the selective activity of Uba1-VHH05. WT Uba1 (*A*) loaded all tested E2 enzymes, regardless of the 6e-tag presence. Uba1-VHH05 (*B*) selectively loaded ubiquitin onto Ube2J1 and the 6e-tagged versions of Ube2R1, Ube2T, and Ube2B, but not the untagged Ube2R1 or Ube2B. Uba1-Nb127D01 (*C*) did not load ubiquitin onto any tested E2s.

Immunoblot analysis using an anti-ubiquitin antibody showed that Uba1–WT efficiently loaded ubiquitin onto all E2 constructs tested (Fig. 3*A*). In addition to thioester formation, incubation with Uba1–WT also resulted in the appearance of di-ubiquitin and higher molecular weight ubiquitin species, consistent with polyubiquitin chain formation. Importantly, Uba1-VHH05 similarly supported ubiquitin loading onto all 6e-tagged E2s while failing to transfer ubiquitin to the untagged Ube2R1 and Ube2B controls (Fig. 3*B*). This confirms that the VHH05-6e pairing is required for E2 recognition and ubiquitin transfer in the absence of the native UFD. In addition to thioester formation, several 6e-tagged E2s, including Ube2B and Ube2R1, still catalyzed the formation of di-ubiquitin and higher molecular weight species in the presence of Uba1-VHH05, mirroring the patterns seen for Uba1-WT.

Both N and C terminally tagged versions of Ube2B were efficiently loaded by Uba1-VHH05, suggesting that positioning of the tag is not essential for functional engagement of these E2s. As expected, the negative control construct Uba1–Nb127D01 showed no detectable ubiquitin transfer to any E2 tested (Fig. 3*C*), underscoring the specificity of the engineered nanobody-epitope interface.

While Uba1-VHH05 retained the ability to support robust thioester formation with appropriately tagged E2s, overall band intensity was noticeably lower than that observed for Uba1-WT. We also observed a decrease in the higher molecular weight species that were detected. Nevertheless, these data show that the engineered system retains key functional characteristics of the native pathway, including the capacity of certain E2s to extend polyubiquitin chains. Although the VHH05-based system is functional and enables selective and flexible engagement of tagged E2s, its transfer efficiency may be reduced relative to WT Uba1.

### Transfer of ubiquitin to E2 is significantly slower for Uba1-VHH05 compared to WT Uba1

**Figure 4:**
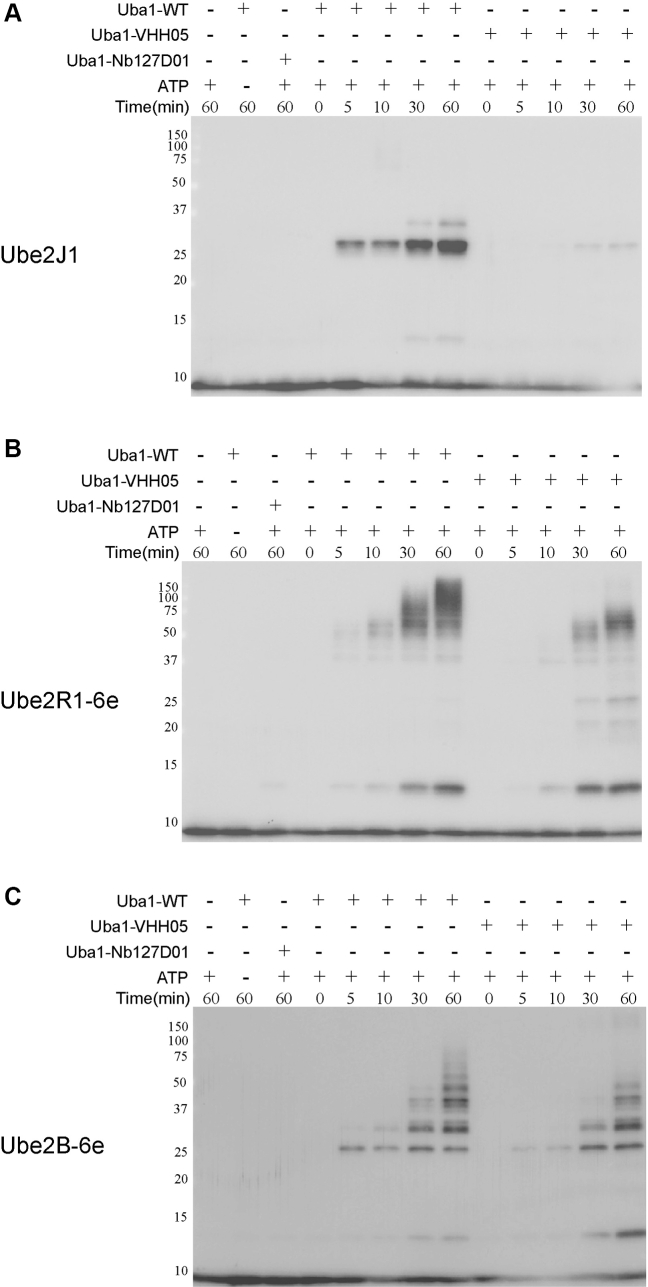
**Uba1-VHH05 has a slower rate of E2-ubiquitin loading compared to Uba1-WT.** Time-course analysis of ubiquitin loading onto Ube2J1 (*A*), Ube2R1-6e (*B*), and Ube2B-6e (*C*) revealed slower thioester formation by Uba1-VHH05 relative to Uba1-WT. Anti-ubiquitin immunoblots illustrate progressive E2-Ub conjugate accumulation over a 0 to 60 min time course. Negative controls lacking ATP or Uba1 show no signal, confirming reaction specificity.

To directly assess differences in the kinetics of ubiquitin transfer between Uba1-WT and Uba1-VHH05, we performed a time-course analysis using Ube2J1, Ube2R1-6e, and Ube2B-6e. Reactions were incubated at 37 °C and sampled at 0, 5, 10, 30, and 60 min. Anti-ubiquitin immunoblotting was used to monitor ubiquitin loading onto these E2s over time. Negative control conditions included the following: (1) omission of Uba1, (2) omission of ATP and finally, (3) inclusion of the control Uba1–Nb127D01 and ATP. None of these controls showed detectable ubiquitin transfer, confirming the specificity and ATP dependency of the reaction (Fig. 4, *A*–*C*, lanes 1–3). SDS-PAGE gel was used to assess sample loading across conditions (Fig. S6). Although loading appeared consistent for most assays (Fig. S6, *A* and *B*), we observed noticeably higher levels of Uba1–VHH05 in the Ube2B–6e time course compared to the corresponding Uba1–WT condition (Fig. S6*C*).

As expected, all three E2s were rapidly loaded with ubiquitin by Uba1-WT, with robust E2-Ub conjugates detectable within 5 min (Fig. 4, *A*–*C*, lanes 4–8). In contrast, Uba1-VHH05 displayed markedly slower kinetics. For Ube2J1, loading by Uba1-VHH05 remained minimal over the time course, with a rather faint signal visible at 60 min (Fig. 4*A*, lanes 9–13). Although Ube2R1-6e loading with Uba1-VHH05 showed stronger signals compared to Ube2J1, a clear delay was seen compared to loading by Uba1-WT (Fig. 4*B*, lanes 9–13 *versus* 4–8, respectively). Strong signals for Ube2R1-Ub conjugates became visible only after 30 min of incubation and did not attain levels similar to those seen with Uba1-WT. Although we also saw a delay for ubiquitin loading onto Ube2B-6e, the effects were less pronounced (Fig. 4*C*). Ubiquitin transfer by Uba1-VHH05 was detectable earlier than for Ube2R1-6e, but still lagged behind the kinetics observed with Uba1-WT (Fig. 4*C*). However, interpretation is complicated by the relatively greater amounts of Uba1-VHH05 in these assays. Nevertheless, the overall trend of delayed kinetics compared to Uba1-WT is clear.

Overall, these results reveal a consistent reduction in the rate of ubiquitin transfer by Uba1-VHH05 compared to WT Uba1 across multiple E2 enzymes. Although VHH05-mediated E2 recognition is sufficient to support selective ubiquitin transfer, the observed kinetic delay thus suggests that the engineered interface is less efficient than the native UFD and may benefit from further optimization for improved catalytic throughput.

## Discussion

Here, we report on the development of an engineered Uba1 ubiquitin-activating enzyme in which the UFD domain is replaced by the VHH05 nanobody (42). This design enables selective charging of any E2 enzyme fused to the cognate 6e-tag, thereby creating a programmable and orthogonal E1-E2 interface that functions within the context of the native ubiquitin conjugation machinery. We show that Uba1-VHH05 retains catalytic activity, can be loaded with both the Ub-Dha probe and WT ubiquitin and can transfer either form of ubiquitin to 6e-tagged E2 enzymes. The negative control (Uba1-Nb127D01) can be loaded with ubiquitin but cannot transfer the ubiquitin to any of the E2s tested, underscoring the specificity of the VHH05-engineered Uba1-E2 interaction. Together, these findings establish a robust platform to dissect the activity and specificity of individual E2s in a native biochemical context.

A key conceptual strength of this system lies in its minimal engineering requirements. Although orthogonal ubiquitin cascades achieve high specificity, they require full redesign of both E1 and E2 enzymes and do not operate within the native ubiquitin context (36). More flexible approaches, such as the tCUbE and E2∼dID systems, allow in-cell or lysate-based profiling of E2 activity, but are restricted to certain classes of E3 enzymes (37, 38). In contrast, our approach only requires the fusion of a short 6e-epitope to the E2 of interest, which allows a single engineered Uba1-VHH05 construct to be used across a wide range of E2s without further modification. Moreover, because ubiquitin is delivered through native enzymatic steps, our system permits interrogation of downstream ubiquitination events without constraining the pathway to a specific class of E3s. This study focuses on reconstituting defined Uba1-E2 pairs within the ubiquitin cascade, but the same modular framework could in principle be extended to test cross-pathway compatibility, for example, by pairing Uba1-VHH05 with tagged E2 enzymes from the SUMO or NEDD8 conjugation pathways. Such experiments are beyond the scope of the present work. Overall, the modularity and scalability of this system make it suitable for probing possible E2-specific roles in ubiquitin- and potentially ubiquitin-like-signaling.

Our data provide strong *in vitro* evidence for selective ubiquitin charging on E2s, but the modularity of our approach could also lend itself to future cell-based applications. Boer *et al.* have shown that the trypanosome brucei Uba1 homologs (TbUba1) are resistant to inhibition by the Uba1 inhibitor TAK-243 (46, 47). Thus, engineering of the UFD domain of TbUba1 in a similar fashion as described here could allow selective charging of tagged E2s in human cells under TAK-243 treatment, effectively removing any interfering effects of endogenous Uba1. This concept offers a promising route toward orthogonal ubiquitination *in vivo*. Precise manipulation of E2 usage in the context of native cellular machinery might thus be possible.

Although the Uba1-VHH05 system is functionally robust, we observed variability in the efficiency of Ub-Dha probe transfer across different E2s. Uba1-VHH05 failed to transfer the probe to 6e-tagged Ube2B, even though it readily supported probe transfer to 6e-tagged Ube2R1 and Ube2J1. Importantly, WT ubiquitin was efficiently transferred to Ube2B-6e, indicating that the effect is probe-specific rather than a general defect in charging. Prior work on a Glycine76Alanine substitution at the C terminus of ubiquitin has shown that even a conservative substitution at this site can be highly detrimental and significantly reduce downstream reactivity (48). Although no direct kinetic comparison has been reported for Ub-Dha, this finding strongly suggests that replacing the terminal glycine with a nonnative residue is likely to decrease transfer efficiency. Indeed, E2-UbDha conversion efficiencies vary across enzymes, with Ube2B appearing less permissive than Ube2J1 (39). However, these results should be interpreted with caution: Ube2B was detected in Ub-Dha pull-down assays, indicating that some interaction occurs, and its apparent low activity in conversion assays may reflect either probe compatibility or reduced intrinsic activity under the assay conditions. A second, and potentially compounding, factor is the geometry of the engineered interface. Both Ube2R1 and Ube2B were tested with C-terminal 6e-tags, but whereas Ube2R1 contains a flexible tail, Ube2B’s C terminus is compact and structurally constrained (Fig. S7) (44). By contrast, the endogenous 6e-tag of Ube2J1 lies in a flexible region, consistent with more efficient probe transfer. Together, these considerations support a “double slowdown” model in which Ub-Dha not only is intrinsically less compatible with Ube2B, but the constrained nanobody-tag docking introduced by Uba1-VHH05 further reduces transfer efficiency. In this scenario, the probe is preferentially retained on Uba1 rather than captured by Ube2B. When WT ubiquitin is used, no such trapping pathway exists, allowing transfer to proceed to Ube2B.

Beyond these probe-specific effects, we also observe a modest decrease in the efficiency of WT ubiquitin transfer. We see consistently slower reaction kinetics relative to WT Uba1. These observations likely reflect differences in the affinity, orientation, or structural flexibility of the engineered nanobody-epitope interface compared to the native Uba1-E2 contacts. We saw a strong effect of linker presence on the functionality of Uba1-VHH05, with the introduction of even a short linker significantly improving probe transfer. Although the position of the tag had little effect on transfer to Ube2B, charging of Ube2J1 with Ub was reduced, despite the presence of its endogenous 6e sequence. In Ube2J1 this tag is not located at its N or C terminus but is embedded. Its accessibility to VHH05 may thus be hindered in the engineered interface and contribute to reduced binding. Further optimization will be necessary. Overall, these findings suggest avenues for continued optimization.

Another factor that may influence downstream efficiency is the high affinity of the VHH05-6e interaction (∼0.15 nM) (42). Under physiological conditions UFD-E2 interactions are transient, allowing for conformational flexibility with the E2 that is required for its release from Uba1, for downstream E3 engagement and, finally, ubiquitin transfer (49, 50, 51, 52). In contrast, a stably bound interface such as the VHH05-6e platform could restrict E2 dynamics or orientation, potentially limiting catalytic throughput in downstream steps. This reduction in rate could result in a mild dominant-negative effect, in which the engineered E1–E2 complex is formed but turns over more slowly due to its stability. Such behavior might not be captured in steady-state loading assays but could become evident in full ubiquitination cascades. A simple titration of Uba1–VHH05 under multiple-turnover conditions could help distinguish between a rate limitation arising from tight complex formation and one intrinsic activity to catalysis. This would provide a useful diagnostic feature for future optimization. Future iterations of this system could therefore benefit from exploring nanobody–epitope pairs with tunable affinities, which may allow fine control over E2 turnover rates and broaden the versatility of the platform.

More broadly, it is important to recognize that the interaction between the UFD of Uba1 and the N-terminal tail of the E2s represents just one component of a larger network of contacts that govern E1-E2 engagement. Previous studies have identified additional contacts that involve the Uba1 catalytic cysteine domains and a tripartite interaction between E2, adenylated ubiquitin, and the crossover loop of Uba1 (29, 53). Both of these alternative sites of engagement were also crucial in mediating efficient ubiquitin transfer. In this context, it will be important to assess whether our engineered interface perturbs these auxiliary binding surfaces. Such insights could offer additional avenues for fine tuning and may help refine the next generation of the approach described here.

A key question for future work is whether this orthogonal E1–E2 pairing can support substrate ubiquitination in a manner comparable to the native cascade. Encouragingly, our *in vitro* assays suggest that E2s retain their characteristic activity within the Uba1-VHH05 framework. Ube2R1 and Ube2B, both known to catalyze E3-independent polyubiquitin chain formation, produced clear di-ubiquitin and higher molecular weight species in our assays (54, 55, 56). The literature does not report any autoubiquitination of Ube2J1, in line with the single Ube2J1∼Ub species we observe. Surprisingly, Ube2T yields some higher molecular weight species despite being predominantly associated with mono-ubiquitination. While unexpected, these bands may reflect multiple mono-ubiquitination events, as prior studies have shown Ube2T can auto-ubiquitinate at multiple lysine residues *in vitro* (57, 58). These findings confirm that Uba1-VHH05 mediated ubiquitin charging preserves key features of endogenous E2 activity and provides a strong foundation for future substrate-level studies.

An important next step will be to determine how selective E2 charging by Uba1-VHH05 influences both E3 engagement and substrate ubiquitination. Although our current data establish that catalytically competent E2∼Ub intermediates are formed, direct measurements of E3 binding and substrate modification have yet to be performed. However, because most RING E3s interact with conserved surfaces on the E2 core domain, we do not anticipate that addition of the 6e-tag to either terminus will substantially perturb these interfaces (59). For E2s in which terminal extensions contribute to E3 recruitment, such as the C-terminal tail of Ube2R1, tag placement can be adjusted accordingly (60, 61, 62). Future work will extend these analyses to test whether selective E2 charging by Uba1-VHH05 supports substrate ubiquitination in more complex environments, including cell extracts or *in vivo* assays, using linkage-specific TUBE pull-downs or diGly-based enrichment to identify modified substrates and characterize ubiquitin-chain architectures. These studies will enable direct comparison between Uba1-WT and Uba1-VHH05 and evaluation of how engineered E1-E2 pairing influences overall pathway throughput.

In conclusion, the Uba1 UFD replacement strategy proposed here offers a versatile and programmable approach for directing ubiquitin conjugation through defined E2s. By decoupling E2 selection from native UFD-mediated interactions, this platform enables precise dissection of E2-specific contributions to ubiquitin signaling. With further optimization and adaptation to cellular models, the system holds promise not only for mechanistic studies but also for the targeted re-engineering of ubiquitin cascades in therapeutic contexts.

## Experimental procedures

### Expression vectors

Human Uba1 was expressed in a PGEX4T vector (#165098, Addgene). Plasmids containing Ube2R1, Ube2B, and Ube2I were a kind gift from the Wade Harper lab, and E2 sequences were subcloned into a pET-SUMO backbone using Gibson assembly (NEB #E2611L). The plasmid containing Ube2J1 (1–197) was previously described (42).

The plasmids expressing Uba1-VHH05 and Uba1-Nb127D01 were generated by replacing the native UFD sequence in the human WT Uba1 vector with either VHH05 or Nb127D01 using Gibson assembly. The 6e-tag was introduced into the E2-containing pET vectors using the Q5 Site-Directed Mutagenesis Kit (NEB #E0554S) and the NEBasechanger for primer design. C-terminal 6e-tags were added to Ube2R1, Ube2T, and Ube2B, while an N terminally tagged version of Ube2B was also generated for comparison (Fig. S2). All constructs were sequence verified prior to expression. To assess the role of linker flexibility, varying numbers of G_4_S linker repeats were introduced between the Uba1 core and the VHH05 nanobody using the same mutagenesis strategy.

### Protein expression and purification

For the cytoplasmic expression of the Uba1 and E2 proteins, BL21(DE3) *E. coli* were grown in Terrific Broth (RPI, T15000) at 37 °C until an *A*_600_ of 0.6 to 0.8 was reached. A final concentration of 1 mM IPTG (Chem-Impex, 00194) was then added and cultures were left to grow O/N either at 16 °C for the Uba1 variants or 30 °C for the E2 constructs (shaking speed 220 rpm). Following overnight incubation, cells were then pelleted at 5,000*g* for 20 min.

Cells containing the Uba1 constructs were resuspended in lysis buffer (50 mM Tris–HCl, pH 8.0, 150 mM NaCl, 10 mM MgCl2, 5% glycerol, 1 mM DTT, cOmplete EDTA-free Protease Inhibitor, and DNAse I) and lysed by passing through an Avestin Emulsiflex C3 homogenizer. Lysate was cleared by centrifugation, and the soluble fraction was run over pre-equilibrated PureCube Glutathione Agarose columns (#32103, Cube Biotech). After washing the beads with lysis buffer, the protein was eluted using lysis buffer containing 20 mM reduced glutathione. The eluates were then concentrated and buffer-exchanged into TBS buffer (50 mM Tris pH 7.5, 150 mM NaCl, 10 mM MgCl_2_, 5% glycerol, and 1 mM DTT) using a 30 kDa cut off filter. The final product was stored at −80 °C.

Cells containing the E2 constructs were pelleted by centrifugation, 15 min at 5000*g*, and resuspended in 30 ml of ice-cold lysis buffer (50 mM Tris–HCl pH 7.5, 150 mM NaCl, cOmplete EDTA-free Protease Inhibitor Cocktail tablet (Sigma-Aldrich, 11873580001), DNAse I (Sigma-Aldrich, 10104159001)). Following a 15 min incubation on ice, cells were lysed using three passages on an Avestin Emulsiflex C3 homogenizer. Crude lysate was clarified by a 30 min centrifugation at 20,000*g*, and protein was purified by binding to Ni-NTA resin (Thermo Fisher Scientific, 88222). Before lysate application the resin was equilibrated using lysis buffer (5 times bed volume) after which the clarified lysate was passed over the column at least twice. The column was then washed twice with 5 times the bed volume using the previously described lysis buffer.

The bound protein was eluted with 250 mM imidazole in 50 mM Tris, 500 mM NaCl, pH 8.0, and then passed through a Fast Protein Liquid Chromatography (FPLC) SEC column (Cytiva HiLoad 16/600 Superdex 75 pg # 28989333) for further purification. Following this process, protein concentrations were measured using the Pierce 660 nm Protein Assay Kit (Thermo Fisher Scientific, 22662) according to the manufacturer's plate protocol.

The ubiquitin Dha probe was synthesized as previously described (39).

## Purified E1 and E2 labeling by the Ub-Dha probe

E2 enzyme (5 μM) and UBA1 enzyme (0.5 μM) in 50 mM Hepes pH 7.5, 100 mM NaCl, 10 mM MgCl_2_, 1 mM DTT, and 10 mM ATP were incubated with biotinylated Ub-Dha probe (2 μM) at 37 °C for described time periods. The reaction was quenched by the addition of sample buffer and the samples were boiled at 95 °C for 7 min. The samples were analyzed by SDS-PAGE and anti-Biotin immunoblot as described under “electrophoresis and immunoblots”.

### 6e-tagged E2 loading with WT ubiquitin

Uba1-variants (1 μM) were incubated with 1 μM of one of the tested E2s and 20 μM of human ubiquitin (R&D Systems #U-100H-10M) in a 100 mM Tris–HCl buffer supplemented with 5 mM MgCl_2_. After set-up of the reaction mixture, 15 μl samples for time point 0 were taken and the ATP was added to a final concentration of 2 mM. Reaction mixtures were incubated at 37 °C with gentle agitation (300 rpm) on an Eppendorf Thermomixer Compact for 1 h after which the second 15 μl sample was taken. Reactions were quenched immediately after sample collection by adding 5 μl of a 4X sample buffer (Thermo Fisher Scientific, NP0007) and boiling at 95 °C for 5 to 10 min. Time-course experiments used the same setup, with 15 μl samples collected after 0, 5, 10, 30, and 60 min of incubation at 37 °C.

### Electrophoresis and immunoblots

Unless otherwise stated, Uba1-loading samples were analyzed on 10% SDS-PAGE gels and E2-loading samples on 12% gels. Gels were stained with InstantBlue Coomassie Protein Stain (ISB1L) (abcam, ab119211).

When analyzing Ub-Dha loading on immunoblot, gels were transferred to a nitrocellulose membrane at 300 mA for 3 h (Bio-Rad, 0.2 μm, 1620112). The membranes were blocked in 5% milk in 1 × PBS for 1 h at room temperature (RT) or O/N at 4 °C. Following blocking, membranes were incubated for 1 h (RT) with horseradish peroxidase-conjugated streptavidin (anti-biotin) in blocking buffer at a 1:10,000 dilution. The membrane was then washed in PBST (0.05% Tween 20) three times. Following washing, the signal was developed using Pierce ECL Western Blotting Substrate (Thermo Fisher Scientific, 32106) and membranes were imaged using the ChemiDoc MP (Bio-Rad, 12003154).

Immunoblot analysis of WT ubiquitin loading onto E2s was performed by transferring the gel to a polyvinylidene difluoride membrane using the Trans-Blot Turbo Transfer System (Bio-Rad, 1704150) with protocols set by manufacturer. Membranes were blocked with blocking buffer (5% bovine serum albumin in TBST (TBS, 0.1% Tween 20)) for a minimum of 1 h at RT or O/N at 4 °C. Following blocking, membranes were rinsed in TBST and incubated for 1 h at RT with anti-ubiquitin horseradish peroxidase (BioLegend # 646303) at a 1:4000 dilution. Membranes were washed three times in TBST for 15 min following the antibody incubation step and developed using the Pierce ECL Western Blotting Substrate. Membranes were imaged using the ChemiDoc MP.

### Structure prediction

The AlphaFold3 server was used to model the structure of Uba1-VHH05 (pTM = 0.86) (44). Structure images for both Uba1 and Uba1-VHH05 were prepared using ChimeraX software (63).

Structural alignment between the Uba1-VHH05 model and the crystal structure of WT Uba1 (PDB: 6DC6) was performed in ChimeraX using the matchmaker command. Alignment was restricted to residues 49 to 954, corresponding to the adenylation domains (IAD and AAD) and the catalytic cysteine half-domains (FCCH and SCCH). The N-terminal tail was excluded from this analysis as it was not defined in the crystal structure. The UFD/VHH05 region was excluded to allow assessment of structural integrity within the Uba1 core. The root-mean-square deviation (RMSD) was calculated over the region described.

## Data availability

All data are contained within the article and supporting information.

## Supporting information

This article contains supporting information.

## Conflict of interest

The authors declare that they have no conflicts of interest with the contents of this article.
