# Supplementary material for: A modular Uba1-nanobody fusion enables selective ubiquitin transfer to tagged E2 enzymes
Source: J Biol Chem. 2025 Nov 5;301(12):110910. doi: 10.1016/j.jbc.2025.110910 (PMC12702329; doi:10.1016/j.jbc.2025.110910)
Supplement: Supplementary Figures [file mmc1.docx]

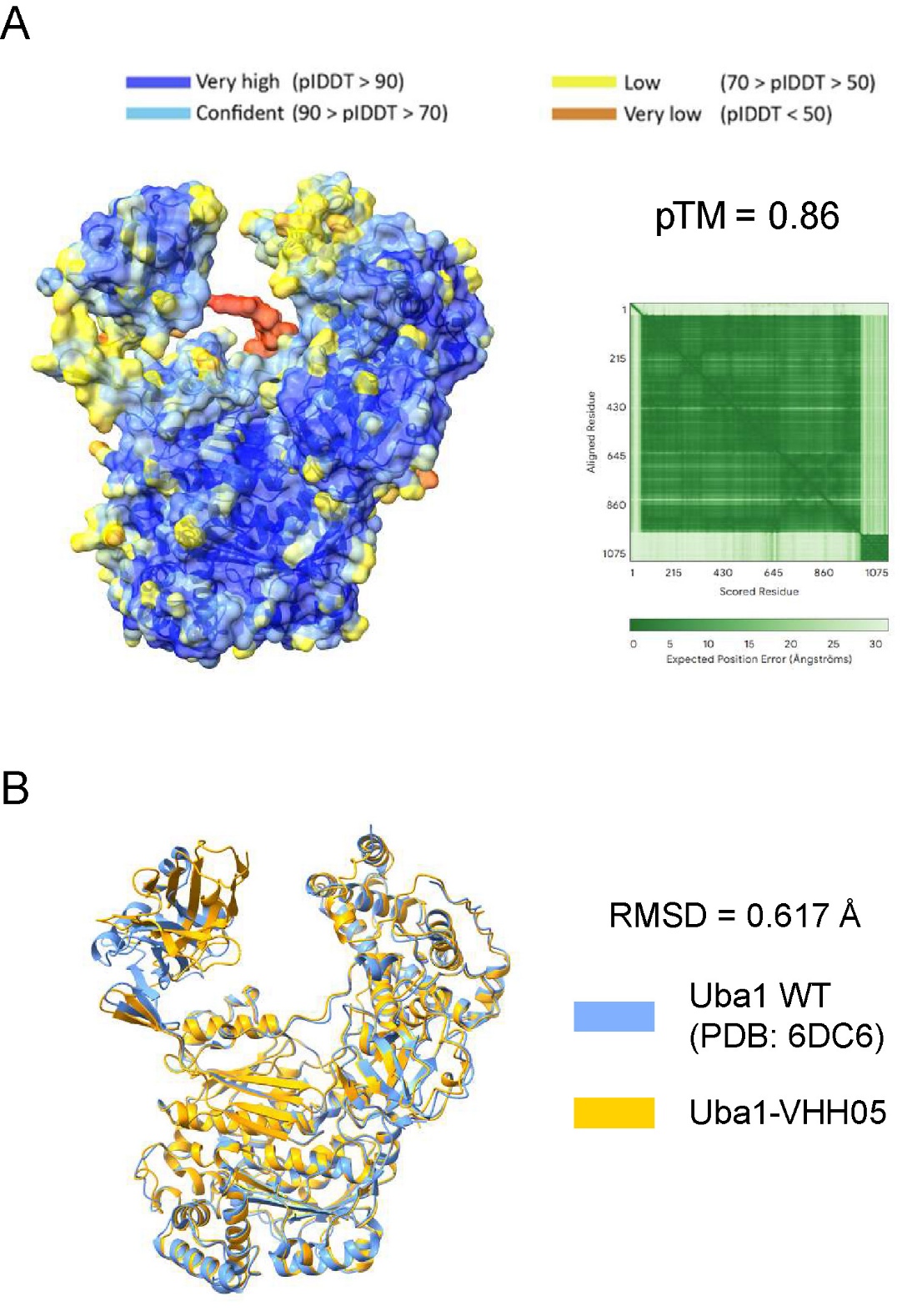


**Supplementary figure 1. AlphaFold3 prediction of the Uba1-VHH05 structure.**

**(A)** The AlphaFold3 predicted structure of Uba1-VHH05. The color coding represents the confidence intervals of predicted regions (pLDDT) and is indicated above the structure. PAE and pTM values are shown to the right. **(B)** The crystal structure of human Uba1 (PDB:6DC6) and the AlphaFold3-predicted model of Uba1-VHH05 were aligned using ChimeraX’s Matchmaker tool. Alignment was restricted to residues 49–954, omitting the UFD and VHH05 regions respectively. The core domains of Uba1 in both structures overlap closely (RMSD = 0.617Å). Structures are shown as cartoons with wild-type Uba1 in blue and Uba1-VHH05 in orange.


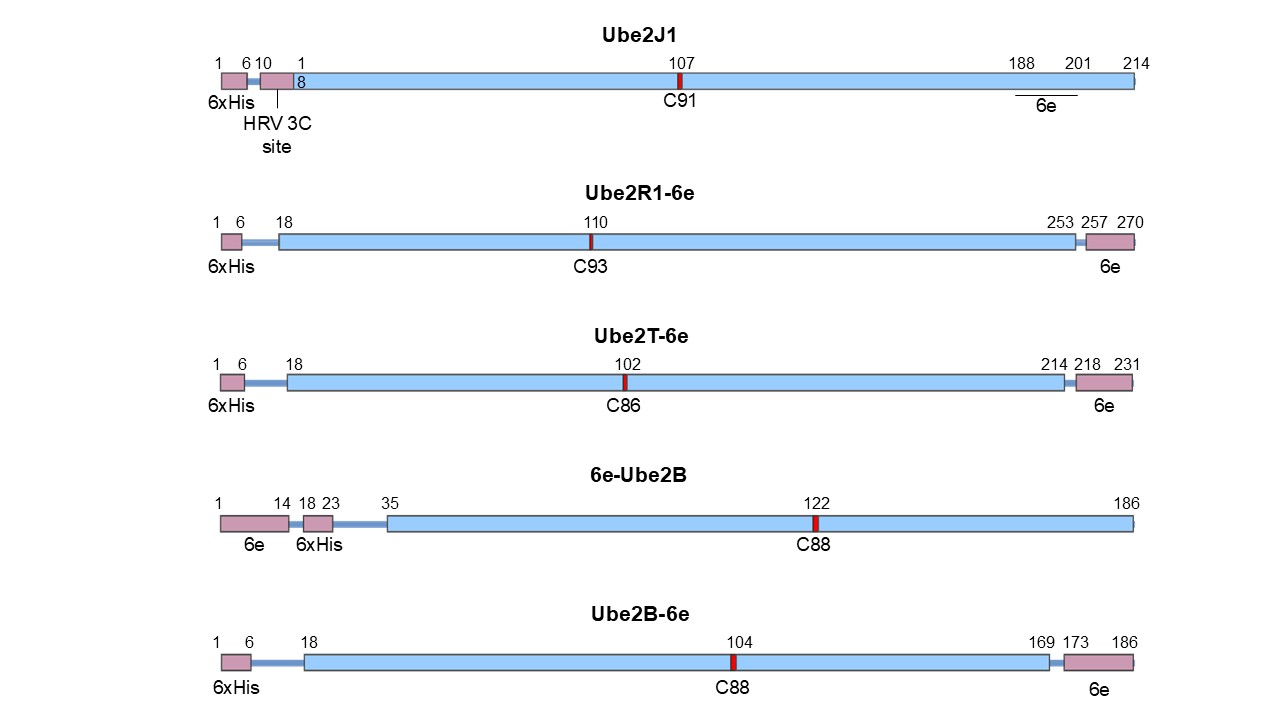


**Supplementary figure 2. Overview of 6e-tagged E2 constructs**

A schematic overview of all produced 6e-tagged E2 constructs. The E2-protein regions are shown in blue. Active site cysteines are marked in red whilst the tags are marked in purple, both are annotated below the sequence. The endogenous 6e-tag in Ube2J1 is marked underneath the sequence. The translation numbering is shown above the sequences.


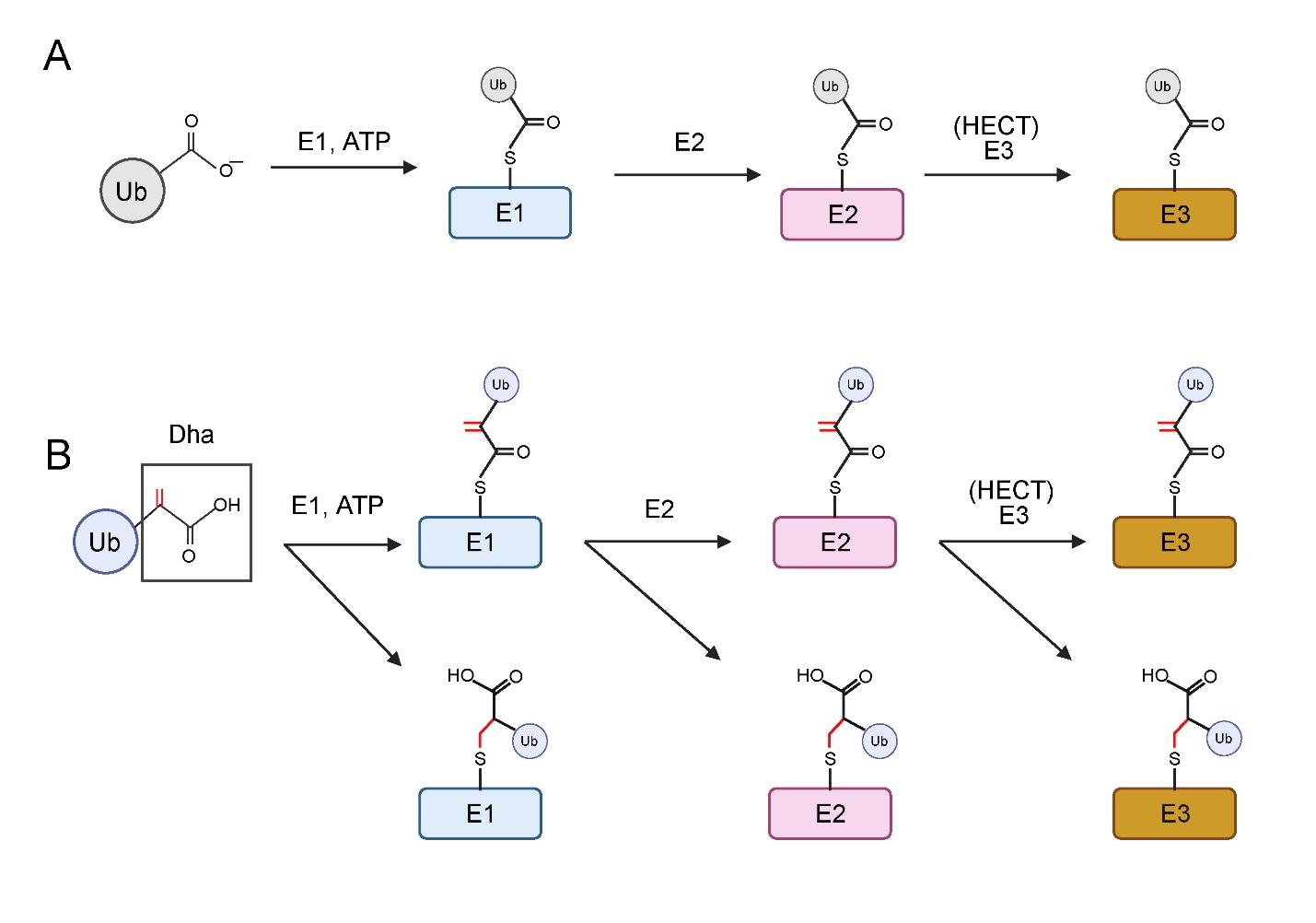


**Supplementary figure 3. Mechanisms of the ubiquitin-dehydroalanine (Ub-Dha) cascading probe**

**(A)** Schematic overview of the native ubiquitin (Ub) pathway. Ubiquitin is activated by the ubiquitin-activating (E1) enzyme in an ATP-dependent reaction which results in a thioester bond between the C-terminal glycine of Ub and the E1 active site cysteine. The Ub is then passed along to a ubiquitin-conjugating (E2) enzyme via a trans-(thio)esterification reaction. Finally, the E2 can work together with a RING or RBR ubiquitin ligase (E3) to ubiquitinate a target (not shown) or transfer the ubiquitin to the active site cysteine of a HECT E3 which can subsequently ubiquitinate a substrate. **(B)** Schematic overview of the Ub-Dha pathway, in which the C-terminal Gly76 is replaced by a Dha residue (outlined). The Ub-Dha probe can either move through the cascade naturally as described (top panel) or it can form an electrophilic intermediate at the activated methylene group (in red) that reacts with the active site cysteine of E1, E2 and HECT E3 enzyme, covalently trapping these enzymes (bottom panel).


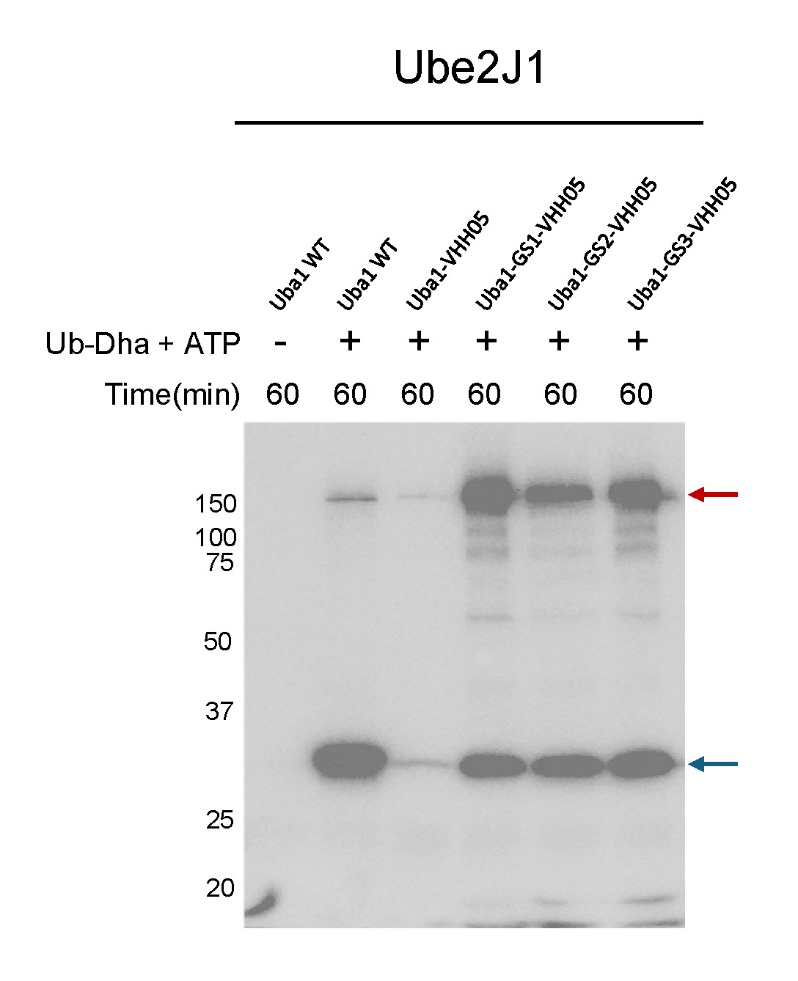


**Supplementary figure 4. Optimization of the Uba1-VHH05 linker length.**
To assess the effect of linker length on the functionality of Uba1-VHH05 an *in vitro* thioester assay was run. Samples containing the Uba1-WT variant or the Uba1-VHH05 variant with either no linker (Uba1-VHH05) or with one (Uba1-GS1-VHH05), two (Uba1-GS2-VHH05) or 3 (Uba1-GS3-VHH05) G4S linker moiety(s) were incubated together with Ube2J1, biotinylated Ub-Dha and ATP and were incubated at 37°C for 1hr after which samples were collected. An immunoblot using streptavidin to detect the movements of the Ub-Dha probe was run to analyze successful ubiquitin loading into Uba1 and Ube2J1. The bands for E1-UbDha are indicated with a red arrow and Ube2J1-UbDha bands are indicated with a blue arrow to the right of the gel.


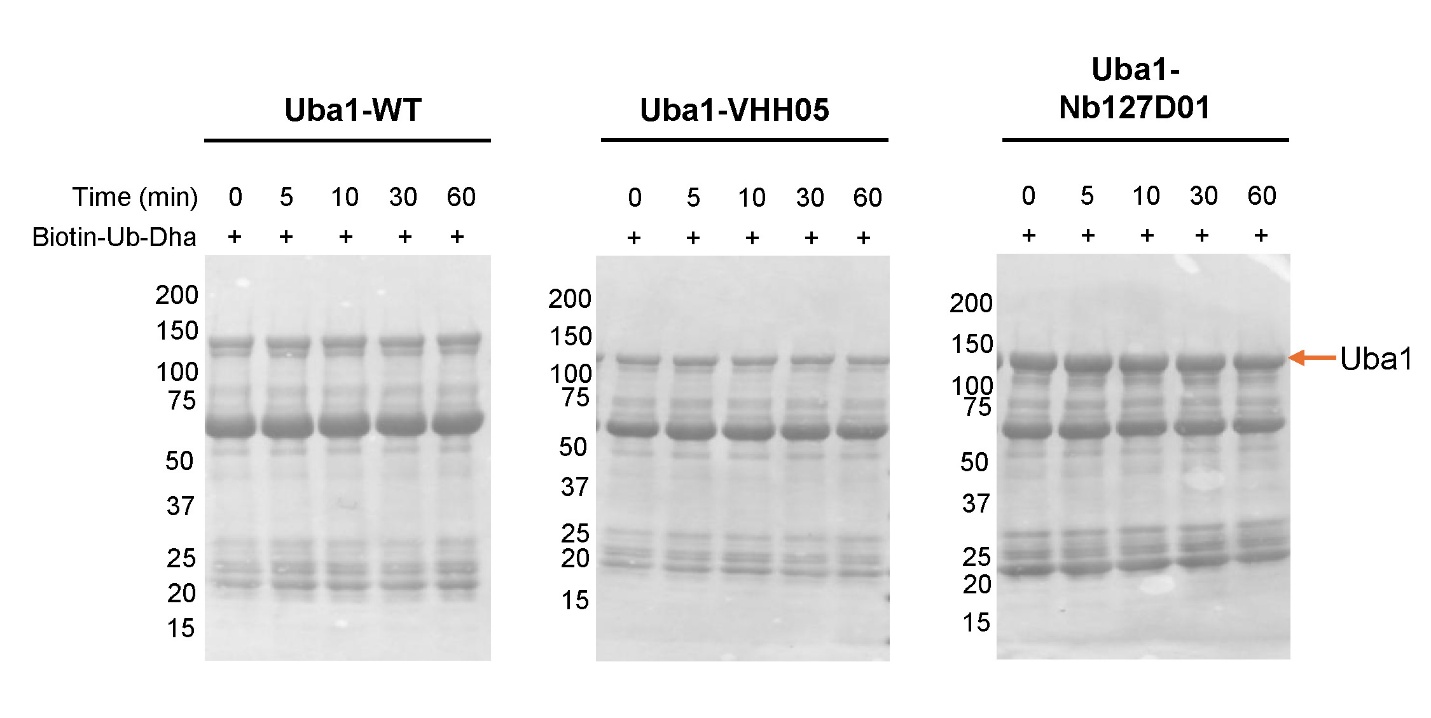


**Supplementary figure 5. Ponceau S staining of blots shown in figure 3**

Ponceau S stained membranes accompanying the streptavidin blots shown in figure 3: **(A)** Uba1-WT **(B)** Uba1-VHH05 **(C)** Uba1-Nb127D01. Uba1 variants are approximately 143 kDa in size and indicated by the orange arrow on the right of the membranes.


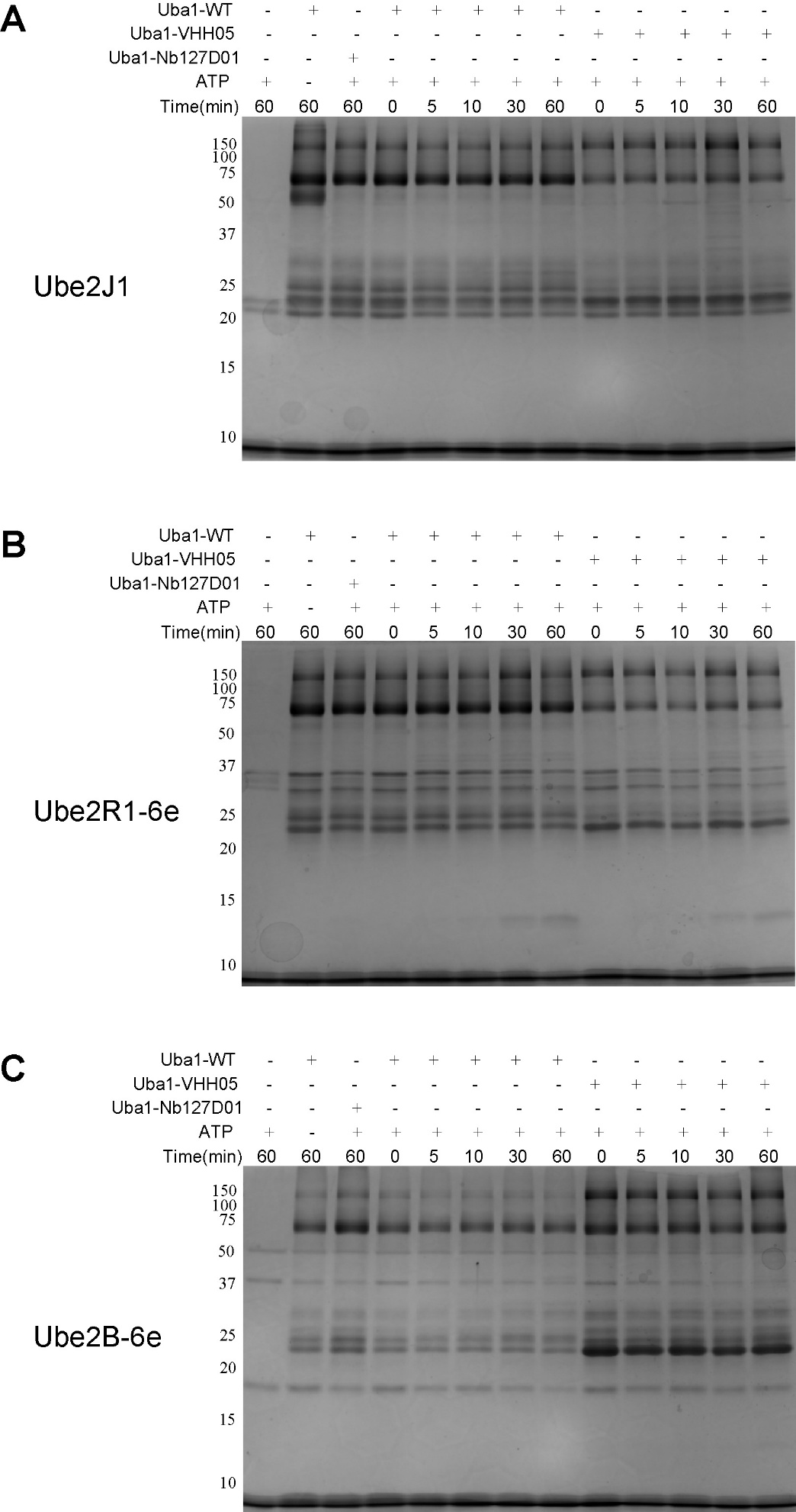


**Supplementary figure 6. Gels accompanying figure 6.**

Coomassie stained non-reducing SDS-PAGE gels accompanying the anti-ubiquitin immunoblots on figure 6: **(A)** Ube2J1 **(B)** Ube2R1-6e **(C)** Ube2B-6e. Uba1 variants are approximately 143 kDa in size and indicated on the right of the gels. Uba1 variants were present in equal amounts in the assays for Ube2J1 and Ube2R1-6e. Uba1-VHH05 was present in higher amounts than Uba1-WT or Uba1-Nb127D01 for the Ube2B-6e assays.


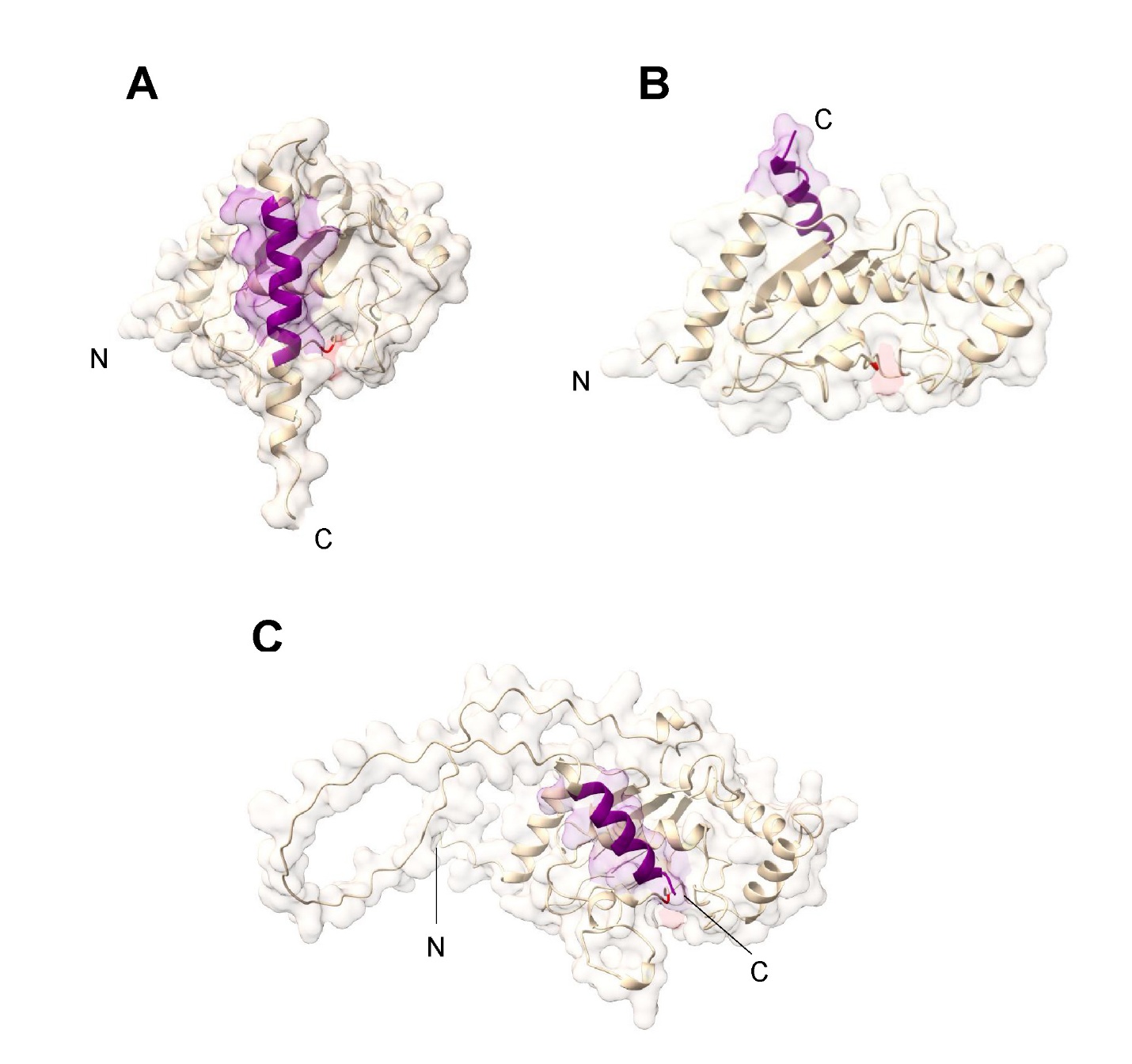


**Supplementary figure 7. AlphaFold predictions of Ube2J1 and C-terminally 6e-tagged Ube2B and Ube2R1**

AlphaFold3 was used to predict the structures of the Ube2J1 construct **(A)** as well as the C-terminally 6e-tagged Ube2B **(B)** and Ube2R1 **(C)**. The active site cysteine is highlighted in red, the 6e-tag is highlighted in purple.
